# Predisposing, Enabling, and Need Factors Associated with Psychotropic Medication and Mental Health Service Use among Children in Out-of-Home Care in the United States: A Scoping Review

**DOI:** 10.3390/ijerph20186769

**Published:** 2023-09-15

**Authors:** Yanfeng Xu, Andrew M. Winters, Nelís Soto-Ramírez, Lauren McCarthy, Gail Betz, Meirong Liu

**Affiliations:** 1 College of Social Work, University of South Carolina, 1512 Pendleton St., Columbia, SC 29208, USA; 2 Kent School of Social Work and Family Science, University of Louisville, Louisville, KY 40292, USA; andrew.winters@louisville.edu; 3 Center for Child and Family Studies, College of Social Work, University of South Carolina, Columbia, SC 29208, USA; sotorami@mailbox.sc.edu; 4 Children’s Hospital Colorado, University of Colorado, Aurora, CO 80045, USA; lauren.mccarthy@cuanschutz.edu; 5 University of Maryland, Baltimore, MD 21201, USA; gbetz@hshsl.umaryland.edu; 6 School of Social Work, Howard University, Washington, DC 20059, USA; meirong.liu@howard.edu

**Keywords:** mental health service, psychotropic medications, out-of-home care, Andersen’s behavioral model of health service use, predisposing, enabling, and need factors

## Abstract

This scoping review aimed to identify predisposing, enabling, and need factors associated with the use of mental health services, including psychotropic medications, among children in out-of-home care in the United States. We searched the PsycInfo, SocINDEX, Medline, and Scopus databases, and 22 studies met inclusion criteria and were systematically analyzed. Among the included studies, 7 studies examined predictors associated with taking psychotropic medications, and 16 examined factors associated with using other mental health services. Significant predisposing, enabling, and need factors associated with greater use of mental health services, including psychotropic medications, were identified. The most frequently identified predisposing factors were child race/ethnicity, age, gender, and maltreatment. Important enabling factors were out-of-home placement type and length of care, and need factors included children’s mental/behavioral problems. The results provide insight into maximizing factors facilitating children’s use of mental health services to address mental health problems of children in out-of-home care. Further, the results imply the importance of the appropriate use of psychotropic medication (e.g., the type and dosage of medications) among children in out-of-home care. The identified factors can inform child welfare agencies and stakeholders on ways to improve access to mental health services and the appropriate use of psychotropic medications among children in out-of-home care in the United States.

## 1. Introduction

### 1.1. Background

In the United States, more than 600,000 children were placed in out-of-home care in 2021, including non-kinship foster, kinship, treatment foster, residential, and group care [1,2]. Children in out-of-home care have higher rates of mental health disorders [3,4,5,6,7]. For instance, 54.4% of foster care alumni in one study had mental health problems compared to 22.1% of the general population [5], leading to a high rate of mental health service needs [8,9,10]. Estimates suggest that children in out-of-home care are 2.5 times more likely to have mental health service needs than the general population [11]. Addressing children’s mental health problems via providing timely and appropriate mental health services to children in out-of-home care would reduce the adverse effects of mental health problems on their short- and long-term well-being. To provide timely and appropriate mental health services, it is important to identify factors associated with children’s use of mental health services in order to eliminate barriers. Mental health services are defined as “any interventions—assessment, diagnosis, treatment, or counseling—offered in private, public, inpatient, or outpatient settings for the maintenance or enhancement of mental health or the treatment of mental or behavioral disorders in individual and group contexts” (para. 1 of [12]). Psychotropic medications are among the treatments many children in out-of-home care receive for their mental health problems. In the present study, we operationalized mental health services as those offered in any setting, such as private, public, inpatient, or outpatient settings, including psychotropic medications provided to children in out-of-home care [13,14].

Despite these children’s high mental health service needs, they have limited access to appropriate mental health services, which may cause an overreliance on psychotropic medications. A prior study indicated that children in foster care are 2–3 times more likely than the general population of children in the community [15] and 2.0–4.5 times more likely than other youth enrolled in Medicaid [16,17] to receive psychotropic medications. There are increased concerns about the overutilization of psychotropic medications among children in out-of-home care, particularly second-generation antipsychotic medication and polypharmacy. An estimated 13–40% of children in out-of-home care receive at least one psychotropic medication [16,18,19,20].

Untreated mental health problems for children in out-of-home care can have detrimental effects on child development, such as increased academic difficulties, high school dropout rates, and risk of homelessness and involvement with the criminal justice system in later life [4,21,22,23,24,25,26]. Similarly, overuse of psychotropic medication is associated with adverse outcomes [27]. Thus, an in-depth understanding of factors associated with using mental health services, including psychotropic medications, will provide insights into how to provide accessible and appropriate mental health services to children in out-of-home care.

### 1.2. Andersen’s Behavioral Model of Health Service Use

**Figure 1:**
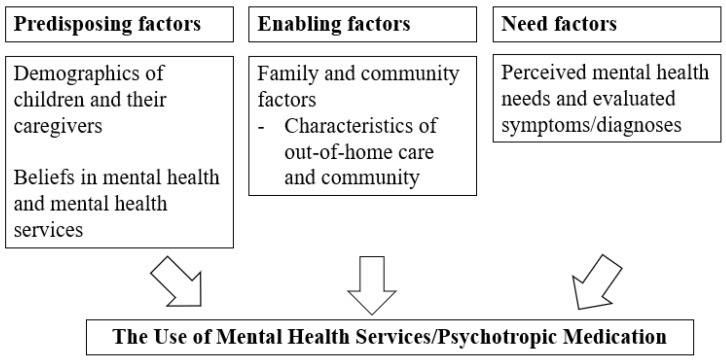
An adapted conceptual framework to understand the use of mental health services among children in out-of-home care.

Andersen’s behavioral model of health service use is a framework for mental health service utilization that distinguishes factors of service use as predisposing, enabling, and need [28,29]. Predisposing factors are individual characteristics, including demographics, social structure, and attitudes or beliefs about medical care, physicians, and disease; enabling factors serve as conditions enabling mental health service utilization; and need factors are perceived needs for mental health services [28]. We used this framework to organize our findings in the current review based on prior applications of this framework to children in out-of-home care [30]. An adapted conceptual framework is provided in Figure 1. For children placed in out-of-home care, predisposing factors include the demographics of children and their caregivers and their beliefs in mental health and mental health services. Enabling factors mainly refer to characteristics of out-of-home care and their neighborhood and community environment. Need factors are their perceived mental health needs and evaluated symptoms and diagnoses.

### 1.3. Study Purpose

Although unmet mental health needs have been a longstanding problem for children in out-of-home care, no prior systematic or scoping review has focused on factors associated with using mental health services, including taking psychotropic medications, among children in out-of-home care. Prior systematic or scoping reviews have primarily focused on mental disorders [31,32] or mental health interventions [6] among children involved in the child welfare system. Identifying factors associated with the use of mental health services among children in out-of-home care would help practitioners and policymakers better serve this population and improve their well-being. To fill this gap in the literature, this scoping review aimed to summarize evidence on factors associated with mental health services and psychotropic medication use among children in out-of-home care guided by Andersen’s model of health service use [28]. Using Andersen’s model of health service use to organize factors is an innovation of this review paper, which further helps identify different layers of factors facilitating or hindering children’s use of mental health services.

## 2. Method

This review followed scoping review methodological guidance [33,34]. The preferred reporting items for systematic reviews and meta-analyses extension for the scoping reviews checklist served as a guideline in reporting this review [35].

### 2.1. Search Strategies

The search strategy was developed through an iterative process in collaboration with a university librarian. The librarian conducted a comprehensive literature search in November 2021. Two key concepts were used to identify studies of interest in databases: mental health service utilization and out-of-home care. The research team searched four databases: PsycInfo, SocINDEX, Medline, and Scopus. The following key terms were employed: (a) mental health service use included search terms such as “mental health treatment”, “mental health programs”, “mental health services”, “counseling”, “therap*”, “therapy”, “behavioral health treatment”, “behavioral health services”, “mental health care”, “mental health personnel”, “mental health practitioner*”, and “mental health professional*”; (b) out-of-home care was searched with terms such as “foster care”, “foster home”, “foster youth”, “foster children”, “foster child*”, “kinship care”, “out of home”, “pre-adoptive home”, “group home”, “congregate care”, “residential treatment”, and “child welfare”. Of note, search terms varied slightly in each database, depending on subject terms, MeSH terms, truncation, and proximity operators when applicable. Literature search results were imported into Covidence [36], an internet-based software program that removes duplicates and facilitates the screening process.

### 2.2. Inclusion and Exclusion Criteria

The included studies met the following criteria: (a) studies that examined factors associated with the use of mental health services and psychotropic medications among children in out-of-home care in the United States and (b) quantitative articles published in peer-reviewed journals between January 2000 and November 2021. Studies were excluded if they (a) examined mental health disorders or problems without a focus on mental health services among children in out-of-home care; (b) evaluated the effects of specific interventions (e.g., cognitive behavioral therapy) on any mental health service use or mental health outcomes; (c) examined substance use disorders and any treatment related to substance misuse or disorder; (d) included children involved in multiple systems (e.g., child welfare, juvenile justice); (e) studied populations not involved with child welfare; (f) combined children in the home and in out-of-home care as a group or did not analyze children in out-of-home care only; (g) used qualitative or mixed methods; (h) did not examine factors associated with the use of mental health services and psychotropic medications (e.g., articles that only examined the prevalence of mental health service use and psychotropic medications); (i) were published as book chapters, dissertations, book reviews, systematic reviews, conceptual papers, and commentaries; and (j) were conducted outside of the United States.

### 2.3. Systematic Searches

**Figure 2:**
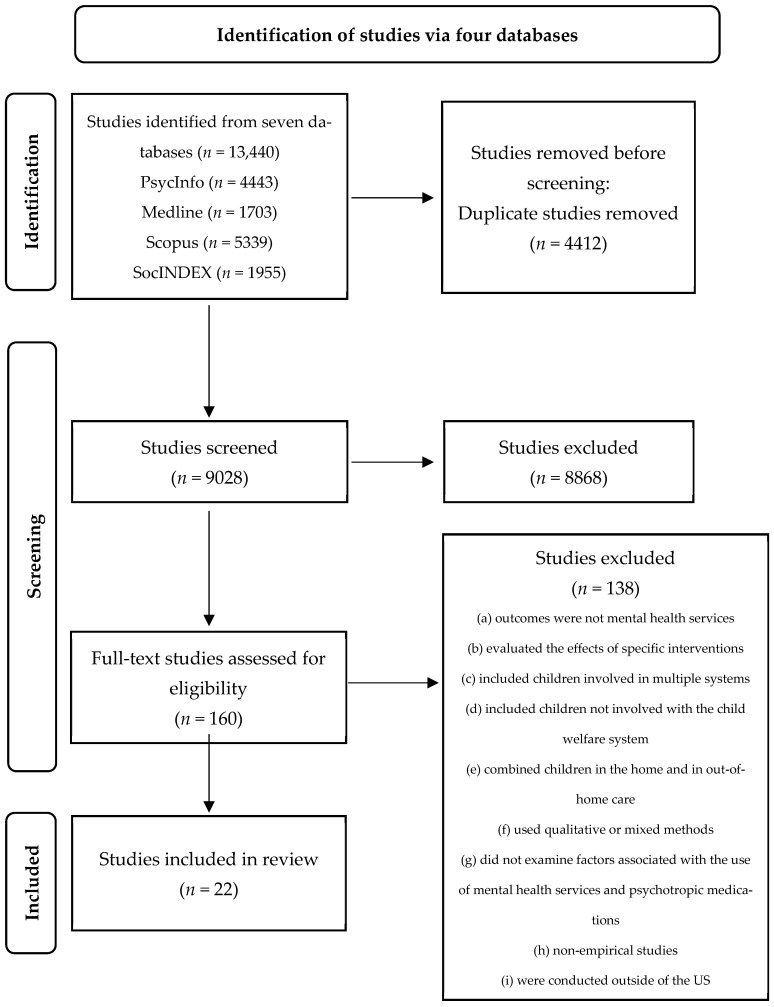
PRISMA chart.

Database searches yielded 13,440 articles (PsycInfo: *n* = 4443; Medline: *n* = 1703; Scopus: *n* = 5339; and SocINDEX: *n* = 1995). After removing 4412 duplicates, 9028 articles were left. The research team screened the 9028 articles’ abstracts and concluded that 160 articles might meet our inclusion criteria. These 160 full-text studies were further screened by reading the full text to conduct an eligibility assessment, and 138 studies were excluded based on one or more of the nine exclusion criteria in Figure 2. This scoping review included 22 studies in the final synthesis.

### 2.4. Data Screening, Extraction, Analysis and Synthesis

Articles were reviewed by the research team using Covidence [36]. The research team members screened study abstracts and titles independently at the abstract screening stage. At the full-text screening stage, the research team independently screened articles, and conflicts were resolved by the first author with input from other research team members.

Extracted data included authors and year of publication, study purpose, data source, basic sample characteristics (see details in Supplementary Table S1), and predisposing, enabling, and need factors associated with psychotropic medication and mental health service use (see details in Table 1). All research team members agreed on the results.

## 3. Results

### 3.1. Study Characteristics

Among the 22 included studies, 8 studies examined predictors associated with psychotropic medications [10,37,38,39,40,43,47,48], whereas 16 studies examined factors associated with the use of mental health services [9,10,38,42,44,45,46,47,49,50,51,52,53,54,55,56]. Of note, two studies examined both outcomes in the same article (i.e., [10,47]).

Most included studies used administrative data to study foster children’s mental health service utilization. The administrative data, including Medicaid claims, mental health claims, child welfare case records, foster care placement records, and closed court cases, were used to answer research questions. Some studies used primary or secondary survey data, such as the National Survey of Child and Adolescent Well-Being [9], self-designed surveys, and interview-assisted surveys, to achieve their research purposes (e.g., [10,47,48,51,56]). In addition, a few studies used data from large intervention studies to describe the use of psychotropic medications (e.g., [40,49]).

Regarding sample characteristics, we report the major characteristics (e.g., age, gender, race, and ethnicity) of children in out-of-home care in the supplementary material. The sample sizes varied from more than 100 (e.g., [49,51,53]) to being in the thousands (e.g., [42,52]). Child age varied across studies, but gender was about evenly distributed. Most children in out-of-home care were White and non-Hispanic.

It is important to note that not all studies referred to predisposing, enabling, and need factors in their conceptual frameworks and analyses. Additionally, we only included significant factors in our synthesis. In terms of positive and negative associations between identified variables and the use of psychotropic medications and mental health services, we use + and − signs to indicate the directions of these associations in Table 1. It is important to note that these positive and negative signs suggest general trends between these variables, but they depend on the reference groups and control variables in the original analyses. Furthermore, other significant factors, such as survey year and study cohort, were not predisposing, enabling, and need factors; thus, we removed them from our results in Table 1. Given the limited number of studies for each outcome, we did not differentiate outcomes when we synthesized results across studies. Results should be interpreted with caution.

### 3.2. Predisposing, Enabling, and Need Factors Associated with the Use of Psychotropic Medications

Because psychotropic medications may have been overused with children in out-of-home care in the past decades, we first analyzed factors associated with this use. Eight articles in this category examined various factors associated with the use of psychotropic medications and were classified into predisposing, enabling, and need factors. 

First, these studies used different methods to operationalize psychotropic medication use, including taking any medication, taking multiple medications, using multiple medication classes, or taking specific types of medication such as attention-deficit/hyperactivity disorder medication, antipsychotics, mood stabilizers, antidepressants, or alpha-agonists. One study examined factors associated with psychotropic medication continuation and discontinuation [47], whereas another examined factors associated with medication use at different times [48]. Because these studies differed in their operationalizations of psychotropic medication use, caution is warranted when interpreting results across studies.

Regarding predisposing factors associated with the use of psychotropic medications, race was a significant predictor, and the directions of the associations were mixed (positive for White and youth of color: [38,47]; and negative for African American, American Indian, and youth of color: [10,43]). McMillen and Raghavan (2009) suggested that youth of color were more likely to discontinue the use of medication, which explained why they had a different direction for this association. Results across studies suggest that White children were more likely to use psychotropic medications than children of color. In addition, we identified that child age (negative for younger than 13 compared to older than 13, ages 5–9 compared to ages 15–17, and based on age at the entrance to foster care: [38,43,47]; positive for ages 6–12 compared to ages 13–21 and continuous age: [40,41]); gender (positive for male: [43]; negative for male: [47]); history of penetrative sexual abuse (no sexual abuse history as reference: [47]); history of physical neglect (no physical neglect history as reference: [47]); and beliefs in the usefulness of medication (positive: [48]) as significant factors. Brenner et al.’s (2014) finding on the relationship between age and use of medication differed from the other studies in that younger children were less likely to use medications than older children. This difference may have been caused by the sample characteristics. Regarding the different findings regarding gender between Glesener et al. (2018) and McMillen and Raghavan (2009), these studies had different outcomes. McMillen and Raghavan (2009) indicated that boys were less likely to continue medication use across the transition period out of foster care compared to girls, whereas Glesener et al. (2018) suggested that boys were more likely to use psychotropic medications than girls in general. Lastly, child maltreatment experience [47] and positive belief in the use of medication [48] were associated with increased use of medications.

We identified enabling factors associated with the use of psychotropic medications. The type of out-of-home care was the most frequently mentioned enabling factor. More specifically, staying in group homes, congregate care, or independent living placements compared to a less restricted out-of-home setting (e.g., foster care and kinship care) increased the likelihood of using medications [10,38,47,48]. Further, some characteristics of out-of-home placement facilitated the use of psychotropic medications. For instance, spending more time in care [41] and foster care specifically [43] was associated with the increased use of medications. In addition, we found that leaving care before age 19 was associated with a higher likelihood of medication discontinuation [47], suggesting that staying in out-of-home care ensures the continuation of medication use. Moreover, Bozzi et al. (2022) found that increased community-level adversity was associated with a lower likelihood of psychotropic medication use.

Regarding need factors, our review identified associations with clinically significant internalizing and externalizing behaviors [38,41], mood disorder [41,47], disruptive behavior disorder [41,47], a disorder in the past 12 months [10], and any mental health or substance use [48]. We also identified using other types of medications (e.g., antidepressants, attention-deficit/hyperactivity disorder medication) as a significant factor because using other psychotropic medications suggests their mental health needs [41]. 

### 3.3. Predisposing, Enabling, and Need Factors Associated with the Use of Mental Health Services

Studies in this category examined factors associated with various mental health services, except for medications, among children in out-of-home care, including number of mental health visits [44], receipt/use of mental health services [45,46,49,50,51,52,53,54,55], dosage [45], number of outpatient mental health visits [46], outpatient mental health services [39,52], in-home counseling or crisis services [39], subsequent crisis visit [42], subsequent psychiatric hospitalization [42], lifetime inpatient psychiatry services [47], lifetime residential or group care [10], lifetime outpatient therapy [10], current residential or group care [10], current outpatient therapy [10], mental health service referral for youth with attention-deficit/hyperactivity disorder or another diagnosis [56], service retention [47], and mental health service discontinuation [47]. Similarly, these studies had slightly different outcomes, which likely affected the direction of associations among key variables.

The first few predisposing factors identified in this review were age [10,39,42,44,45,46,50,52,53,54,55] and gender [44,45,46,47,55]. General trends across studies indicated that younger and male children were more likely to receive mental health services than older and female children. Regarding the association between race and ethnicity and outcomes of interest, results were mixed across studies [10,39,42,44,45,46,47,52,54,55]. Specifically, a few studies suggested that African Americans were more likely than their White counterparts to receive mental health services [39,44,55]. Similarly, two studies found that Latinos were more likely to receive mental health services than Whites [45,52]. This may suggest a higher prevalence of mental health issues among children of color, which may be related to the fact that they are more likely to be diagnosed or pathologized by mental health providers. The remaining studies indicated that youth of color, particularly Latino and other races and ethnicities, were less likely to receive mental health services (e.g., outpatient mental health services, lifetime inpatient psychiatry, lifetime outpatient therapy, current residential or group care, and current outpatient therapy) than their White counterparts [10,42,44,46,54], which may be related to barriers to receiving mental health services among racial and ethnic minorities.

In addition, we identified additional predisposing factors related to the use of mental health services, including child maltreatment history [10,46,47,49,50,51,54,55], parental substance abuse [52], caregiver absence [44,46], history of juvenile detention [45,47], release from state custody prior to age 19 [47], each 6 month period of earlier discharge [47], removal due to a voluntary agreement [50], and foster parent education [56]. The directions of these relationships were as expected and presented in Table 1.

The most important enabling factor associated with foster children’s use of mental health services was the type of out-of-home care [9,39,44,45,46,47,49,50,51,52]. The general finding indicated that staying in more restricted out-of-home care (e.g., group home, residential care, congregate care) was associated with more mental health service use than in more family-like settings (e.g., kinship care). Other enabling factors related to out-of-home care and the neighborhood or community were the number of placement changes [44,45], length of care [45,51,56], first placement [50], reason for case closure (emancipating from foster care vs. other reasons) [54], referral to mental health services [53], and race composition density by county [50]. Specifically, having more placement changes and staying in care longer were associated with more mental health service use [44,45]. Experiencing the first placement was associated with a lower likelihood of engaging in mental health services [50]. Villagrana (2017) suggested that youth emancipated from foster care were less likely to use mental health services, whereas foster children achieving permanency were more likely to use mental health services. Villagrana (2010) found that having a referral to mental health services compared to having no referral to mental health services was associated with more mental health service use. Lastly, being White in a low or medium-population-density county was associated with an increased likelihood of using mental health services, whereas being African American, Asian, or Pacific Islander in a high-population-density county and being Latino in a county with low- or medium-population density was associated with a decreased probability of service receipt [50].

Need factors identified in this review included foster children’s internalizing and externalizing behavioral problems [39,44,46], mental disorders (e.g., psychotic, mood, or disruptive behavior disorders [44], mental illness (e.g., bipolar, anxiety, attention-deficit/hyperactivity, conduct, impulsive, or adjustment disorder) [45,50,52], anxiety [45,51], disorder in lifetime or past 12 months [10], psychological well-being [51], physical health problems [52], and level of comorbidity [45,52,56]. In general, having more mental or behavioral problems was positively associated with mental health service utilization, and one study found that having posttraumatic stress disorder in the past year was associated with decreased odds of mental health service retention [47]. In addition, we identified that prior outpatient, day treatment, crisis visit, and inpatient services [42] were associated with increased subsequent use of mental health services (e.g., subsequent crisis visits or psychiatric hospitalization).

## 4. Discussion and Conclusions

### 4.1. Discussion

This study systematically reviewed 22 studies on factors associated with psychotropic medication and other mental health service use among children in out-of-home care in the United States. A total of 8 studies examined factors associated with psychotropic medication use, and 16 explored factors associated with mental health service use; 2 looked at both. Most studies utilized administrative data; however, some studies utilized primary or secondary survey/intervention data. The results of the review were organized around predisposing, enabling, and need factors as outlined by Andersen’s behavioral model of health service use [28]. Although we list these factors here, we must acknowledge that they are selective, depending on what factors were originally considered by the included articles.

Results regarding predisposing factors were consistent for both psychotropic medication use and mental health service use; however, the nature of the relationship sometimes differed depending on the sample characteristics, operationalizations of independent and dependent variables, and control variables. Most studies suggested that being male was positively associated with more psychotropic medication use, and being female was positively associated with more mental health service use. These differences may indicate gender differences in accessing mental health services, including psychotropic medications. In addition, being older was positively associated with psychotropic medication use except for one study [40], whereas most studies found that younger age was positively associated with mental health service use (e.g., [9,10,52]). This may indicate that as youths aged, a common practice was simply prescribing medications instead of appropriate treatments, which may suggest that older youths have received less effective therapeutic interventions, leading to increased psychotropic medication use. Further, race and ethnicity were consistently associated with both psychotropic medication and mental health service use. However, the nature of the relationship between service use and racial and ethnic identity also differed across studies. A general trend suggested that racial and ethnic minorities use more medications and have less access to other mental health services. However, a more in-depth understanding of racial and ethnic disparities in accessing mental health services is needed. Our review also identified other predisposing factors associated with psychotropic medication and mental health service use, such as child maltreatment history and other childhood adversities, foster parents’ characteristics, and beliefs in the usefulness of medications.

Regarding significant enabling factors identified in this review, child placement was the most significant factor. Children staying in more restrictive settings were more likely to receive more mental health services. For instance, staying in congregate, group, or residential care was an enabling factor positively associated with mental health service use (e.g., [39,45]). In contrast, placement in kinship care was an enabling factor found to have a negative association with increased mental health service use, apart from one study [52]. In this review, we also identified other enabling factors that facilitate or hinder children’s use of mental health services, as previously discussed. Finally, factors related to perceived need associated with both psychotropic medication and mental health service use included clinically significant internal and external behavioral concerns, diagnosed mood or anxiety disorder, and any mental health disorder in the past 12 months. The only unique factors related to perceived need positively associated with receipt of mental health services were prior service receipt and psychosis [42], and one study found that a diagnosis of posttraumatic stress disorder was negatively associated with mental health service use [47].

### 4.2. Implications for Practice

This scoping review’s findings on predisposing, enabling, and need factors associated with psychotropic medications and mental health services among children in out-of-home care in the United States generated some implications for practice.

First, this review identified different effects of predisposing factors, such as the child’s race and ethnicity, age, and gender, on the use of mental health services and psychotropic medications among children in out-of-home care. This result suggests the importance of screening and identifying children in out-of-home care who experience mental health challenges, focusing on reducing racial and ethnic, age, and gender disparities in accessing mental health services. Further, this review sheds light on the associations between types of child maltreatment and mental health service utilization that deserve attention from social workers and other mental health providers. In addition, this review identified other predisposing factors, such as foster parent education and perceptions of the usefulness of medications, suggesting that we need to promote these factors to improve the use of psychotropic medications among children in out-of-home care.

Second, this review generated implications regarding enabling factors that give children in out-of-home care easy access to psychotropic medication and mental health services. Our review suggests that children in group homes and institutional care are more likely to receive constant mental health services than children in non-kinship foster care and kinship care. Hence, social workers and other mental health professionals can eliminate barriers that result in children in family-like settings having less access to mental health services. Further, social workers and other mental health professionals can advocate for providing foster parents, particularly kinship caregivers, with the educational, financial, and instrumental resources they need to support their children’s mental health [45]. Additionally, studies found that an early exit from care was associated with increased psychotropic medication use and decreased mental health service use [47]. This may indicate that youth who exit care may be losing vital support necessary to connect with mental health services. Because the use of mental health services is related to Medicaid or other insurance coverage, continued insurance coverage, particularly Medicaid coverage, is vital for young adults aging out of foster care to receive needed mental health services and psychotropic medications. Advocating for Medicaid expansion for young adults aging out of foster care is needed.

Lastly, this review provides some insights into children’s needs. It seems that children with greater needs were more likely to have access to mental health services and greater use of psychotropic medications. However, in practice, the neediest children may not have access to these needed services; if they receive these services, they may not receive the most appropriate ones [57]. Thus, it is important to advocate for children who need these services desperately but do not have the capacity to navigate the child welfare and mental health systems.

In summary, this review suggests the importance of combined efforts at national, state, and local levels to address these disparities by applying thorough and consistent screening and identification of children in out-of-home care with mental health challenges. Given the high need for mental health services among children in out-of-home care, collaboration and relationships among child welfare, Medicaid, and mental health service providers need to be strengthened to improve the efficient and effective delivery of mental health services. As such, a multisector approach to mental health services for children in out-of-home care is needed. For instance, the provision of accessible, evidence-based, and culturally responsive interventions for using these mental health services and psychotropic medications is warranted.

### 4.3. Limitations

Despite this review’s contributions to the literature, some limitations should be noted when interpreting findings. First, our search was limited to quantitative peer-reviewed journal articles published in English between 2000 and 2021 in the United States. This excluded studies published in other languages in other countries, earlier than 2000, and in non-peer-reviewed journals or gray literature. Additional studies from other countries may have a different cultural viewpoint that would add valuable knowledge regarding predisposing, enabling, and need factors related to psychotropic medications and mental health services. Also, mixed-methods, qualitative, intervention, and evaluation studies were excluded, which may have overlooked additional insights on this topic among children in foster care. Second, all studies reviewed had different designs, but we did not distinguish any differences when reviewing their findings. Longitudinal studies can ascertain more reliable causal relationships than cross-sectional studies. Cross-sectional studies are bound by several limitations, such as selection and recall biases and lack of evidence of temporal relationships. The direction of the effect between predictors and outcomes might be bidirectional. Thus, it is important to be cautious about generalizing this scoping review’s findings because we mixed results from cross-sectional studies with those from longitudinal studies. Lastly, we used a qualitative synthesis approach to summarize our findings but did not conduct a meta-analysis, which may further limit the generalizability of our findings.

### 4.4. Directions for Future Research

Based on critiques of included studies and gaps in the literature, this scoping review indicates future research directions to understand the use of psychotropic medications and mental health services among children in out-of-home care. First, more research is greatly needed to identify a comprehensive list of predisposing, enabling, and need factors associated with psychotropic medication and mental health service use among children in out-of-home care. Researchers need to explore buffers and mechanisms that facilitate the use of psychotropic medications and mental health services. Particularly, policies and procedures may influence mental health services for children in out-of-home care, yet they likely vary by state and county. Research is needed to understand state- and county-level factors and other organizational and systemic factors that might influence the receipt of mental health services. Second, researchers could focus on integrating the perspectives of youth, caseworkers, caregivers, and practitioners to understand under what conditions youth may disengage from mental health services. Third, studies should investigate how the mental health needs of children in out-of-home care are being differentially addressed across race and ethnicity. Relatedly, researchers could investigate the mental health needs and experiences of youth identifying as mixed race or other in child welfare contexts. In addition, researchers need to integrate disparities in gender, age, and race and ethnicity into this research line. Finally, more longitudinal studies should be conducted to examine the trajectories of mental health services and psychotropic medication use among children in out-of-home care.

### 4.5. Conclusions

This scoping review summarized various predisposing, enabling, and need factors associated with the use of mental health services, including psychotropic medications, among children in out-of-home care. It identified mixed evidence regarding predisposing factors associated with the use of mental health services and psychotropic medications. Gender, age, and racial and ethnic disparities still exist in access to these services. Regarding enabling factors, the type of out-of-home care determined the likelihood of mental health service receipt and use of psychotropic medications. Regarding need factors, this review showed that children with greater mental health needs were more likely to use psychotropic medications and receive mental health services. The results of this review suggest the importance of eliminating barriers that hinder children’s access to mental health services and facilitating predisposing, enabling, and need factors that would promote more use of mental health services for these children. Furthermore, it is warranted to engage state child welfare agencies, practitioners, foster care advocates, and legislators in ensuring equal access to mental health services.

## Figures and Tables

**Table 1:** Predictors associated with mental health service or psychotropic medication use (*N* = 22).

Author, Year	Predictors Associated with Mental Health Service or Psychotropic Medication Use	
Bozzi et al., 2022 [37]	**DV**: Psychotropic use **Predisposing**: n/a **Enabling**: Community adversity – **Need**: n/a	
Breland-Noble et al., 2004 [38]	**DV**: Taking any medication **Predisposing**: White + Younger than 13 (ref: >13) − **Enabling**: Group home (ref: therapeutic foster care) + **Need**: Clinical range on externalizing CBCL subscale + Clinical ranges for both externalizing and internalizing subscales +	**DV**: Taking multiple medications **Predisposing**: Younger − **Enabling**: n/a **Need**: Clinical ranges on both externalizing and internalizing CBCL scores +
Breland-Noble et al., 2005 [39]	**DV**: Outpatient mental health **Predisposing**: Age − **Enabling**: Group home (ref: therapeutic foster care) + **Need**: CBCL total score +	**DV**: In-home counseling or crisis services **Predisposing**: African American (ref: White) + **Enabling**: n/a **Need**: CBCL total score +
Brenner et al., 2014 [40]	**DV**: Any psychotropic medication use **Predisposing**: Aged 6–12 (ref: 13–21) + **Enabling**: n/a **Need**: n/a	**DV**: ADHD medication **Predisposing**: Aged 6–12 (ref. 13–21) + **Enabling**: n/a **Need**: n/a
DosReis et al., 2014 [41]	**DV**: Antipsychotic **Predisposing**: Age + **Enabling**: n/a **Need**: Mood disorder (ref: no diagnosis) + Antidepressant (ref: no use) + ADHD medication (ref: no use) + **DV**: ADHD medication **Predisposing**: Age + **Enabling**: n/a **Need**: Disruptive behavior disorder +	**DV**: Mood stabilizer **Predisposing**: n/a **Enabling**: n/a **Need**: Disruptive behavior disorder − **DV**: Antidepressant **Predisposing**: Age + **Enabling**: n/a **Need**: Internalizing disorder +
Fawley-King and Snowden, 2012 [42]	**DV**: Subsequent crisis visit **Predisposing**: Hispanic (ref: White) − Aged 6–11 (ref: 12–18) − **Enabling**: n/a **Need**: Prior outpatient treatment (ref: none) + Day treatment (ref: none) + Inpatient stay (ref: none) +	**DV**: Subsequent psychiatric hospitalization **Predisposing**: Aged 6–11 (ref: 12–18) − Placement change prior to hospitalization (ref: none) + **Enabling**: n/a **Need**: Prior outpatient treatment (ref: none) + Day treatment (ref: none) + Inpatient stay (ref: none) +
Glesener et al., 2018 [43]	**DV**: Any psychotropic medication **Predisposing**: Aged 5–9 (ref: 15–17) − Time in foster care + **Enabling**: n/a **Need**: n/a **DV**: Antidepressant **Predisposing**: Aged 5–9 (ref: 15–17) − Time in foster care + **Enabling**: n/a **Need**: n/a **DV**: ADHD **Predisposing**: Aged 5–9 (ref: 15–17) − American Indian (ref: White) − Male + Time in foster care + **Enabling**: n/a **Need**: n/a	**DV**: Alpha-agonists **Predisposing**: Male + **Enabling**: n/a **Need**: n/a **DV**: Antipsychotic **Predisposing**: Aged 5–9 − Male + **Enabling**: n/a **Need**: n/a **DV**: Multiple medication classes **Predisposing**: Aged 5–9 − African American (ref: White) − American Indian (ref: White) − Male + Time in foster care + **Enabling**: n/a **Need**: n/a
James et al., 2004 [44]	**DV**: Number of mental health visits **Predisposing**: Female (ref: male) − Age at entry into out-of-home care + African American (ref: White) + Hispanic (ref: White) − Other (ref: White) − Caretaker absence − **Enabling**: Number of placement changes + Episodes in kinship care − **Need**: Behavior problems +	
Kim et al., 2021 [45]	**DV**: Receipt of MH service **Predisposing**: Age − Female (ref: male) − Latino (ref: White) + Juvenile justice involvement + **Enabling**: Placement instability + Placement type (ref: none) Foster care + Kinship care − Group home + Institution + Months in dependent care + **Need**: n/a	**DV**: Dosage of MH service **Predisposing**: Age − Female (ref: male) − Juvenile justice involvement − **Enabling**: Placement type (ref: none): Foster care + Kinship care + Group home + Institution + Months in dependent care + **Need**: Psychotic disorder + Mood disorder + Disruptive behavior disorder + Anxiety disorder + Adjustment disorder + Other disorder + Comorbidity +
Leslie et al., 2000 [46]	**DV**: Number of outpatient mental health visits **Predisposing**: Age + Latino (ref: White) − Asian or other (ref: White) − Male (ref: female) + Sexual abuse (ref: no) − Caregiver absence (ref: no) − **Enabling**: Kinship care only (ref: foster care only) − Kinship or foster care (ref: foster care only) − **Need**: Total CBCL ≥ 60 (ref: < 60) +	
Leslie et al., 2004 [9]	**DV**: Use of outpatient mental health service **Predisposing**: Aged 2–3 (ref: 11+) − Aged 6–10 (ref: 11+) − Physical neglect (ref: no) − Sexual abuse (ref: no) + **Enabling**: Group or residential care (ref: foster care) + **Need**: CBCL score ≥ 64 (ref: below 64) +	
McMillen and Raghavan, 2009 [47]	**DV**: Service retention **Predisposing**: Male (ref: female) − History of juvenile detention − Release from state custody prior to age 19 − **Enabling**: n/a **Need**: Posttraumatic stress disorder − **DV**: MH service discontinuation **Predisposing**: Youth of color + History of physical neglect − Each 6-month period of earlier discharge + **Enabling**: Congregate care (ref: other, with family, non-kinship foster family, and living more independently) − **Need**: n/a	**DV**: Psychotropic medication discontinuation **Predisposing**: Youth of color (ref: White) + History of penetrative sexual abuse (ref: no) − **Enabling**: Congregate care (ref: other, with family, non-kinship foster family, and living more independently) − Left care aged 17 to 17.5 (ref: did not leave before 19) + Left care aged 17.5 to 18 (ref: did not leave before 19) + Left care aged 18 to 18.5 (ref: did not leave before 19) + Left care aged 18.5 to 19: (ref: did not leave before 19) + **Need**: History of disruptive behavioral disorder + **DV**: Continued medication use across transition out of foster care **Predisposing**: Male (ref: female) − History of physical neglect – **Enabling**: n/a **Need**: n/a
McMillen et al., 2004 [10]	**DV**: Lifetime inpatient psychiatry **Predisposing**: Youth of color − Age at entrance to foster care system − Physical abuse (ref: no) + Sexual abuse (ref: no) + **Enabling**: n/a **Need**: Lifetime disorder (ref: no) + **DV**: Lifetime residential or group care **Predisposing**: Youth of color + Age at entrance to foster care system − **Enabling**: n/a **Need**: Lifetime disorder (ref: no) + **DV**: Lifetime outpatient therapy **Predisposing**: Youth of color − Age at entrance to foster care system − Physical abuse + Physical neglect − Sexual abuse + **Enabling**: n/a **Need**: n/a	**DV**: Current psychotropic medication **Predisposing**: Youth of color − Age at entrance to foster care system − **Enabling**: Congregate care (ref: non-kinship family foster home or living more independently) + **Need**: Disorder in past 12 months + **DV**: Current residential or group care **Predisposing**: Youth of color − Physical abuse − **Enabling**: n/a **Need**: Disorder in past 12 months + **DV**: Current outpatient therapy **Predisposing**: Youth of color − **Enabling**: n/a **Need**: Disorder in past 12 months +
Park et al., 2019 [48]	**DV**: Psychotropic medication use at age 17 **Predisposing**: n/a **Enabling**: Congregate care (ref: nonrelative foster home) + Independent living (ref: nonrelative foster home) + **Need**: Any mental health issue or substance use (ref. no diagnosis) +	**DV**: Psychotropic medication use at age 19 **Predisposing**: Used medication, agreed that good things outweigh bad things (ref: no meds) + Used medication, neutral that good things outweigh bad things (ref: no meds) + Used medication, disagreed that good things outweigh bad things (ref: no meds) + **Enabling**: n/a **Need**: n/a
Petrenko et al., 2011 [49]	**DV**: Receiving mental health services at Time 1 **Predisposing**: History of physical or sexual abuse (ref: other maltreatment type) + **Enabling**: Nonrelative foster care (ref: kinship) + **Need**: n/a	**DV**: Receiving mental health services at Time 2 **Predisposing**: n/a **Enabling**: n/a **Need**: Received recommendation for new services +
Pullmann et al., 2018 [50]	**DV**: Service receipt within 4 months among those screening above criteria **Predisposing**: Age at time of removal + African American in high-density county − Asian or Pacific Islander in high-density county − Physical abuse (ref: neglect) + Sexual abuse (ref: neglect) + **Enabling**: Relative caregiver (ref. all other) − Nonrelative caregiver (ref. all other) − **Need**: Bipolar + Anxiety + ADHD, conduct, impulsive disorder + Adjustment disorder +	**DV**: Service receipt within 4 months among those screening below criteria **Predisposing**: Age at time of removal + Physical abuse (ref: neglect) + Sexual abuse (ref: neglect) + **Enabling**: n/a **Need**: ADHD, conduct, impulsive disorder + Adjustment disorder + **DV**: Continued engagement in behavioral health or evidence-based services among those above criteria **Predisposing**: Physical abuse + Removal due to voluntary agreement (ref: court order) − **Enabling**: Caucasian in low-density county − African American in medium-density county − Native American in low-density county − First placement – **Need**: n/a
Shin et al., 2005 [51]	**DV**: Mental health service use **Predisposing**: Child abuse history (ref. no) + Time in care + **Enabling**: Nonrelative foster care (ref: kinship) + **Need**: Anxiety + Psychological well-being +	
Swanke et al., 2016 [52]	**DV**: Outpatient mental health services **Predisposing**: Hispanic (ref: non-Hispanic) + Physical abuse + Parental substance abuse + Age − **Enabling**: Placement in kinship care − **Need**: Physical health problems + Adjustment reaction disorder + Attention deficit disorder + Conduct disorder + Comorbidity −	
Villagrana, 2010 [53]	**DV**: Mental health utilization **Predisposing**: Aged 11–16 (ref: 5–10) + Sexual abuse (ref. neglect) + **Enabling**: Referral to mental health services (ref. not referred) + **Need**: n/a	
Villagrana et al., 2017 [54]	**DV**: Mental health service use during foster care **Predisposing**: Aged 18 (ref: 17) + Aged 19 (ref: 17) + **Enabling**: Other case closure reason (ref: court ordered) + Emancipation case closure (ref: court ordered) + **Need**: n/a	**DV**: Mental health service use after foster care **Predisposing**: Physical abuse (ref: neglect) + Sexual abuse (ref: neglect) + Latino (ref: White) − **Enabling**: Other case closure (ref: court ordered) + Emancipation case closure (ref: court ordered) + **Need**: n/a
Yampolskaya et al., 2017 [55]	**DV**: Mental health service use **Predisposing**: Male (ref: female) + Age + African American (ref: White) + Other race and ethnicity (ref: White) + Sexual abuse (ref: threatened harm) + Neglect (ref: threatened harm) + Maltreatment chronicity + **Enabling**: n/a **Need**: n/a	
Zima et al., 2000 [56]	**DV**: Mental health service referral for ADHD **Predisposing**: Foster parent education + Time in care – **Enabling**: n/a **Need**: n/a	**DV**: Mental health service referral for other diagnosis **Predisposing**: Foster parent education + **Enabling**: n/a **Need**: Level of comorbidity +

Note. ADHD = attention-deficit/hyperactivity disorder; CBCL = Child Behavior Checklist; DV = dependent variable. Plus or minus signs after factors indicate positive or negative associations, respectively.

## Data Availability

Data sharing not applicable.

## Supplementary Materials

The following supporting information can be downloaded at https://www.mdpi.com/article/10.3390/ijerph20186769/s1. Table S1: Included Study Characteristics (*N* = 22).

## References

[1] Child Welfare Information Gateway Out-of-Home Care. https://www.childwelfare.gov/topics/outofhome/.

[2] U.S. Department of Health and Human Services (2022). Trends in Foster Care and Adoption: FY 2012–2021. https://www.acf.hhs.gov/cb/report/trends-foster-care-adoption.

[3] Anctil T.M., McCubbin L.D., O’Brien K., Pecora P. (2007). An evaluation of recovery factors for foster care alumni with physical or psychiatric impairments: Predictors of psychological outcomes. Child. Youth Serv. Rev..

[4] Courtney M.E., Dworsky A. (2006). Early outcomes for young adults transitioning from out-of-home care in the USA. Child Fam. Soc. Work.

[5] Pecora P.J., Jensen P.S., Romanelli L.H., Jackson L.J., Ortiz A. (2009). Mental health services for children placed in foster care: An overview of current challenges. Child Welf..

[6] Hambrick E.P., Oppenheim-Weller S., N’zi A.M., Taussig H.N. (2016). Mental health interventions for children in foster care: A systematic review. Child. Youth Serv. Rev..

[7] Pecora P.J., Williams J., Kessler R.C., Downs A.C., O’Brien K., Hiripi E., Morello S. (2003). Assessing the Effects of Foster Care: Early Results from the Casey National Alumni Study.

[8] Harman J.S., Childs G.E., Kelleher K.J. (2000). Mental health care utilization and expenditures by children in foster care. Arch. Pediatr. Adolesc. Med..

[9] Leslie L.K., Hurlburt M.S., Landsverk J., Barth R., Slymen D.J. (2004). Outpatient mental health services for children in foster care: A national perspective. Child Abus. Negl..

[10] McMillen J.C., Scott L.D., Zima B.T., Ollie M.T., Munson M.R., Spitznagel E. (2004). Use of mental health services among older youths in foster care. Psychiatr. Serv..

[11] Burns B.J., Phillips S.D., Wagner H.R., Barth R.P., Kolko D.J., Campbell Y., Landsverk J. (2004). Mental health need and access to mental health services by youths involved with child welfare: A national survey. Child Adolesc. Psychiatry.

[12] American Psychological Association Mental Health Services. https://dictionary.apa.org/mental-health-services.

[13] Burns B.J., Costello E.J., Angold A., Tweed D., Stangl D., Farmer E.M., Erkanli A. (1995). Children’s mental health service use across service sectors. Health Aff..

[14] Horwitz S.M., Hurlburt M.S., Goldhaber-Fiebert J.D., Heneghan A.M., Zhang J., Rolls-Reutz J., Fisher E., Landsverk J., Stein R.E. (2012). Mental health services use by children investigated by child welfare agencies. Pediatrics.

[15] Raghavan R., Zima B.T., Andersen R.M., Leibowitz A.A., Schuster M.A., Landsverk J. (2005). Psychotropic medication use in a national probability sample of children in the child welfare system. J. Child Adolesc. Psychopharmacol..

[16] Kutz G.D. (2011). Foster Children: HHS Guidance Could Help States Improve Oversight of Psychotropic Prescriptions.

[17] Raghavan R., Brown D.S., Allaire B.T., Garfield L.D., Ross R.E. (2014). Medicaid expenditures on psychotropic medications for maltreated children: A study of 36 states. Psychiatr. Serv..

[18] Leslie L.K., James S., Monn A., Kauten M.C., Zhang J., Aarons G. (2010). Health-risk behaviors in young adolescents in the child welfare system. J. Adolesc. Health.

[19] Raghavan R., Alexandrova A. (2015). Toward a theory of child well-being. Soc. Indic. Res..

[20] Raghavan R., McMillen J.C. (2008). Use of multiple psychotropic medications among adolescents aging out of foster care. Psychiatr. Serv..

[21] Bender K., Yang J., Ferguson K., Thompson S. (2015). Experiences and needs of homeless youth with a history of foster care. Child. Youth Serv. Rev..

[22] Cusick G.R., Havlicek J.R., Courtney M.E. (2012). Risk for arrest: The role of social bonds in protecting foster youth making the transition to adulthood. Am. J. Orthopsychiatry.

[23] Harris M.S., Jackson L.J., O’Brien K., Pecora P.J. (2009). Disproportionality in education and employment outcomes of adult foster care alumni. Child. Youth Serv. Rev..

[24] Iglehart A.P., Becerra R.M. (2002). Hispanic and African American youth: Life after foster care emancipation. J. Ethn. Cult. Divers. Soc. Work..

[25] Lee J.S., Courtney M.E., Tajima E. (2014). Extended foster care support during the transition to adulthood: Effect on the risk of arrest. Child. Youth Serv. Rev..

[26] Villegas S., Rosenthal J.A., O’Brien K., Pecora P. (2011). Health outcomes for adults in family foster care as children: An analysis by ethnicity. Child. Youth Serv. Rev..

[27] Camp A.R. (2010). A mistreated epidemic: State and federal failure to adequately regulate psychotropic medications prescribed to children in foster care. Temple Law Rev..

[28] Andersen R., Newman J.F. (1973). Societal and individual determinants of medical care utilization in the United States. Milbank Meml. Fund Q. Health Soc..

[29] Lederle M., Tempes J., Bitzer E.M. (2021). Application of Andersen’s behavioural model of health services use: A scoping review with a focus on qualitative health services research. BMJ Open.

[30] Xu Y., Jedwab M., Soto-Ramirez N., Weist M.D. (2023). The use of mental health services among children in kinship care: An application of Anderson’s behavioral model for health services use. J. Public Child Welf..

[31] Bronsard G., Alessandrini M., Fond G., Loundou A., Auquier P., Tordjman S., Boyer L. (2016). The prevalence of mental disorders among children and adolescents in the child welfare system: A systematic review and meta-analysis. Medicine.

[32] Engler A.D., Sarpong K.O., Van Horne B.S., Greeley C.S., Keefe R.J. (2022). A systematic review of mental health disorders of children in foster care. Trauma Violence Abus..

[33] Arksey H., O’Malley L. (2005). Scoping studies: Towards a methodological framework. Int. J. Soc. Res. Methodol..

[34] Levac D., Colquhoun H., O’Brien K.K. (2010). Scoping studies: Advancing the methodology. Implement. Sci..

[35] Tricco A.C., Lillie E., Zarin W., O’Brien K.K., Colquhoun H., Levac D., Moher D., Peters M.D.J., Horsley T., Weeks L. (2018). PRISMA extension for scoping reviews (PRISMA-ScR): Checklist and explanation. Ann. Intern. Med..

[36] Veritas Health Innovation Covidence Systematic Review Software. https://www.covidence.org.

[37] Bozzi L.M., Shah P., DosReis S. (2022). Community adversity and utilization of psychotropic medications among children in foster care. J. Behav. Health Serv. Res..

[38] Breland-Noble A.M., Elbogen E.B., Farmer E.M., Dubs M.S., Wagner H.R., Burns B.J. (2004). Use of psychotropic medications by youths in therapeutic foster care and group homes. Psychiatr. Serv..

[39] Breland-Noble A.M., Farmer E.M., Dubs M.S., Potter E., Burns B.J. (2005). Mental health and other service use by youth in therapeutic foster care and group homes. J. Child Fam. Stud..

[40] Brenner S.L., Southerland D.G., Burns B.J., Wagner H.R., Farmer E.M. (2014). Use of psychotropic medications among youth in treatment foster care. J. Child Fam. Stud..

[41] DosReis S., Tai M.H., Goffman D., Lynch S.E., Reeves G., Shaw T. (2014). Age-related trends in psychotropic medication use among very young children in foster care. Psychiatr. Serv..

[42] Fawley-King K., Snowden L.R. (2012). Relationship between placement change during foster care and utilization of emergency mental health services. Child. Youth Serv. Rev..

[43] Glesener D., Anderson G., Li X., Brown J., Amell J., Regal R., Ferguson D. (2018). Psychotropic medication patterns for American Indian children in foster care. J. Child Adolesc. Psychopharmacol..

[44] James S., Landsverk J., Slymen D.J., Leslie L.K. (2004). Predictors of outpatient mental health service use—The role of foster care placement change. Ment. Health Serv. Res..

[45] Kim M., Barnhart S., Garcia A.R., Jung N., Wu C. (2021). Changes in mental health service use over a decade: Evidence from two cohorts of youth involved in the child welfare system. Child Adolesc. Soc. Work. J..

[46] Leslie L.K., Landsverk J., Ezzet-Lofstrom R., Tschann J.M., Slymen D.J., Garland A.F. (2000). Children in foster care: Factors influencing outpatient mental health service use. Child Abus. Negl..

[47] McMillen J.C., Raghavan R. (2009). Pediatric to adult mental health service use of young people leaving the foster care system. J. Adolesc. Health.

[48] Park K., Okpych N.J., Courtney M.E. (2019). Psychotropic medication use and perceptions of medication effects among transition-age foster youth. Child Adolesc. Soc. Work J..

[49] Petrenko C.L., Culhane S.E., Garrido E.F., Taussig H.N. (2011). Do youth in out-of-home care receive recommended mental health and educational services following screening evaluations?. Child. Youth Serv. Rev..

[50] Pullmann M.D., Jacobson J., Parker E., Cevasco M., Uomoto J.A., Putnam B.J., Benshoof T., Kerns S.E.U. (2018). Tracing the pathway from mental health screening to services for children and youth in foster care. Child. Youth Serv. Rev..

[51] Shin S.H. (2005). Need for and actual use of mental health service by adolescents in the child welfare system. Child. Youth Serv. Rev..

[52] Swanke J.R., Yampolskaya S., Strozier A., Armstrong M.I. (2016). Mental health service utilization and time to care: A comparison of children in traditional foster care and children in kinship care. Child. Youth Serv. Rev..

[53] Villagrana M. (2010). Mental health services for children and youth in the child welfare system: A focus on caregivers as gatekeepers. Child. Youth Serv. Rev..

[54] Villagrana M. (2017). Racial/ethnic disparities in mental health service use for older foster youth and foster care alumni. Child Adolesc. Soc. Work J..

[55] Yampolskaya S., Sharrock P.J., Clark C., Hanson A. (2017). Utilization of mental health services and mental health status among children placed in out-of-home care: A parallel process latent growth modeling approach. Child Psychiatry Hum. Dev..

[56] Zima B.T., Bussing R., Yang X., Belin T.R. (2000). Help-seeking steps and service use for children in foster care. J. Behav. Health Serv. Res..

[57] Dashiell-Earp C., Zlotnik S. (2011). Psychotropic medications and children in foster care. Policy Pract..

