# Supplementary material for: Predisposing, Enabling, and Need Factors Associated with Psychotropic Medication and Mental Health Service Use among Children in Out-of-Home Care in the United States: A Scoping Review
Source: Int J Environ Res Public Health. 2023 Sep 15;20(18):6769. doi: 10.3390/ijerph20186769 (PMC10531290; doi:10.3390/ijerph20186769)
Supplement: Supplementary file 1 [file ijerph-20-06769-s001.zip › ijerph-2494516-supplementary.pdf]

**Table S1**. Included Study Characteristics (*N* = 22).

| Author, year | Study purpose | Data source | Sample characteristics |
|---|---|---|---|
| Bozzi et al., 2022 | To examine the association between community adversity and psychotropic use among children in foster care in one U.S. state | Integrated foster care and Medicaid administrative databases, Kids Count, U.S. census, and Area Health Resource File in 2014 | *N* = 4,334<br>**Age:**<br>5–9: 28%<br>10–14: 29%<br>15–18: 43%<br>**Gender:**<br>Male: 49%<br>Female: 51%<br>**Race:**<br>Black: 63%<br>White: 33%<br>Other: 4% |
| Breland-Noble et al., 2004 | To examine the use of psychotropic medications among youths in residential community-based placements | Statewide study among children who stayed in therapeutic foster care (June 1999 through May 2001) and group homes (January through June 2001) | *N* = 304<br>Therapeutic foster care: *n* = 184<br>Group home: *n* = 120<br>**Age:**<br>*M* = 14.1<br>**Gender:**<br>Group home:<br>Female: 74%<br>Male 26%<br>Therapeutic foster care<br>Female: 87%<br>Male: 13%<br>**Race:**<br>White: 58%<br>African American: 42% |
| Breland-Noble et al., 2005 | To examine patterns of multisector service use for youth in therapeutic foster care and group homes | Federally funded statewide study of therapeutic foster care (June 1999 through May 2001) and group home care (February 2001 through July 2001) | *N* = 304<br>Therapeutic foster care: *n* = 184<br>Group home: *n* = 120<br>**Therapeutic foster care:**<br>**Age:**<br>*M* = 14.1<br>**Gender:**<br>Male: 74%<br>Female: 26%<br>**Race:**<br>White: 55%<br>African American: 40%<br>Other: 5%<br>**Group home:**<br>**Age:**<br>*M* = 14.3<br>**Gender:**<br>Male: 87%<br>**Race:**<br>White: 49%<br>African American: 47% |

| | | | |
|---|---|---|---|
| | | | Other: 4% |
| Brenner et al., 2014 | To describe the use of psychotropic medications, including polypharmacy and an indicator of questionable polypharmacy among youth in treatment foster care | Randomized trial that tested the effectiveness of an enhanced treatment foster care model conducted in usual care agencies between 2003 and 2008 in a southeastern state | *N* = 247<br>**Age:**<br>*M* = 13.0<br>**Gender:**<br>Female: 45%<br>Male: 55%<br>**Race:**<br>White: 32%<br>African American: 58%<br>Other: 10% |
| DosReis et al., 2014 | To investigate changes in the prevalence of psychotropic medication use for each year increase in age from 3 to 6 among children in foster care and to examine time-varying odds of longer duration of use by each year of age | Child welfare administrative records and state department of mental health claims data during 2009–2011 | *N* = 1,491<br>**Age:**<br>≤ 3: 69%<br>4: 10%<br>5: 12%<br>6: 10%<br>**Gender:**<br>Male: 52%<br>Female: 48%<br>**Race:**<br>White: 20%<br>Black: 77%<br>Other: 2% |
| Fawley-King & Snowden, 2012 | To (a) confirm a relationship between placement change during a foster care episode and use of emergency mental health care, specifically crisis services and psychiatric hospitalization; and (b) differentiate between placement change's impact on emergency mental health care and emergency mental health care's impact on placement change | Medi-Cal paid claims and foster care placement records between 1998 and 2001 | *N* = 19,351<br>**Gender:**<br>Female: 54%<br>Male: 46%<br>**Race and ethnicity:**<br>White: 34%<br>Black: 23%<br>Asian: 3%<br>Hispanic: 38%<br>Other: 2%<br>**Age at start of episode:**<br>6–11: 59%<br>12–18: 41% |
| Glesener et al., 2018 | To assess the effects of race, age, sex, and time spent in foster care on rates of psychotropic medication use for children in foster care in 2012 | Electronic records through county social service and state Medicaid in 2012 | *N* = 626<br>**Age:**<br>0–4: 39.8%<br>5–9: 23%<br>10–14: 19.5%<br>15–17: 17.7%<br>**Gender:**<br>Female: 45%<br>Male: 55%<br>**Race and ethnicity:**<br>White: 53%<br>American Indian: 36%<br>African American: 11% |
| James et al., 2004 | To examine the relationship between placement change and outpatient mental health | Medicaid claims data and children's social services records in California | *N* = 570<br>**Age at entry to care:**<br>*M* = 7.3 |

| | | | |
|---|---|---|---|
| | service use among children in foster care | | **Gender:**<br>Male: 45.6%<br>Female: 54.4%<br>**Race and ethnicity:**<br>White: 45.3%<br>Hispanic: 20.4%<br>African American: 28.4%<br>Other: 6.0% |
| Kim et al., 2021 | To examine rates and predictors of MH service use over time among child welfare involved youth. | Child welfare and juvenile justice system data and Medicaid-funded behavioral health claims data from a large mid-Atlantic city of the United States | *N* = 8,225<br>**Age:**<br>*M* = 15.08<br>**Gender:**<br>Male: 48.25%<br>Female: 51.75%<br>**Race and ethnicity:**<br>Caucasian: 10.49%<br>African American: 75.90%<br>Latino: 11.91%<br>Other: 1.69% |
| Leslie et al., 2000 | To determine factors influencing outpatient mental health service use by children in foster care | Medicaid, San Diego County Mental Health Management Information System, and California Department of Social Services | *N* = 480<br>**Age:**<br>1–3: 31.3%<br>4–5: 19.2%<br>6–11: 38.3%<br>12–17: 11.3%<br>**Gender:**<br>Male: 44.8%<br>Female: 55.2%<br>**Race and ethnicity:**<br>White: 44.2%<br>African American: 27.7%<br>Latino: 22.5%<br>Asian or other: 5.6% |
| Leslie et al., 2004 | To determine factors influencing the use of outpatient mental health services provided by mental health professionals for children in foster care | National Survey of Child and Adolescent Well-Being | *N* = 462<br>**Age:**<br>2–3: 19.6%<br>4–5: 12.5%<br>6–10: 37.3%<br>11+: 30.5%<br>**Gender:**<br>Female: 49.1%<br>Male: 50.9%<br>**Race and ethnicity:**<br>White: 36.7%<br>African American: 39.4%<br>Hispanic: 16.3%<br>Other: 7.7% |
| McMillen & Raghavan, 2009 | To assess and predict changes in mental health service use as older youth leave the foster care system | Youth interviewed at age 19 and enrolled in a longitudinal study of older youth from the foster care system | *N* = 325<br>**Age:**<br>19<br>**Gender:**<br>Female: 61%<br>Male: 39% |

| | | | |
|---|---|---|---|
| | | | **Race and ethnicity:**<br>59% person of color<br>41% White |
| McMillen et al., 2004 | To examine (a) lifetime, 12-month, and current mental health service use among older youths in the foster care system and (b) variations in mental health care by race, gender, maltreatment history, living situation, and geographic region | Survey with 406 foster youth via telephone | *N* = 406<br>**Age:**<br>*M* = 16.99<br>**Gender:**<br>Female: 56%<br>Male: 44%<br>**Race and ethnicity:**<br>White: 44%<br>African American: 51%<br>Mixed race: 4%<br>American Indian: 1%<br>Asian: < 1%<br>Latino: < 1%<br>Middle Eastern: < 1% |
| Park et al., 2019 | To (a) examine the prevalence of psychotropic medication use over time; (b) evaluate youths' perceptions of the benefits of medication, and (c) assess associations between medication use and behavioral health problems | California Youth Transitions to Adulthood Study, a panel study of a cohort of transition-age foster youth in California | *N* = 611<br>**Age:**<br>16.75–17.75<br>**Gender:**<br>Female: 60%<br>Male: 40%<br>**Race and ethnicity:**<br>White: 17.6%<br>Black: 17.3%<br>Hispanic: 47.4%<br>Mixed race: 15.2%<br>Other: 2.4% |
| Petrenko et al., 2011 | To determine if recommendations from mental health and educational screening evaluations were related to service implementation for youth in out-of-home care | 171 maltreated youth in out-of-home care and their caregivers who participated in the randomized controlled trial of the Fostering Healthy Futures program from 2002 to 2007 | *N* = 171<br>**Age:**<br>*M* = 10.38<br>**Gender:**<br>Male: 49.7%<br>Female: 50.3%<br>**Race and ethnicity:**<br>White: 44.4%<br>African American: 28.1%<br>Hispanic or Latino: 49.7%<br>Native American: 8.8%<br>Asian: 2.3%<br>Hawaiian or other Pacific Islander: 2.3% |
| Pullmann et al., 2018 | To explore the case flow and trajectories of children and youth as they enter the child welfare system, are screened, and receive services for mental health | Administrative records from all 8,636 children and youth aged 3 to 17 who entered out-of-home care for 30 days or more between July 2010 and July 2014 in Washington | *N* = 8,636<br>**Age:**<br>*M* = 8.45<br>**Gender:**<br>Female: 51.5%<br>Male: 48.5%<br>**Race and ethnicity:**<br>White: 53.8%<br>African American: 8.0%<br>Hispanic: 16.2%<br>Asian or Pacific Islander: 2.2% |

| | | | |
|---|---|---|---|
| | | | Native American: 3.6%<br>Multiracial: 15.9% |
| Shin et al., 2005 | To explore the need for and actual use of mental health services by adolescents in the child welfare system | Face-to-face interviews with foster adolescents, Medicaid Paid Claims Longitudinal Database, and a Midwest state's child welfare administrative database | *N* = 113<br>**Age:**<br>*M* = 17.5<br>**Gender:**<br>Male: 46%<br>Female: 54%<br>**Race and ethnicity:**<br>African American: 70%<br>White: 25%<br>Asian, Native American, or unknown: 7% |
| Swanke et al., 2016 | To compare mental health service receipt and time to receive mental health services between youth in kinship care versus youth in nonkinship foster homes | Administrative child welfare data and Medicaid data from fiscal year 2007 through fiscal year 2010 in Florida | *N* = 36,543<br>**Age:**<br>*M* = 4.79<br>**Gender:**<br>Male: 50%<br>Female: 50%<br>**Race and ethnicity:**<br>Non-Hispanic White: 48%<br>African American: 38%<br>Hispanic: 13%<br>Other: 1% |
| Villagrana, 2010 | Examine characteristics that predict referral to mental health services, and whether referral was a significant predictor of service utilization | Closed court cases between March and December 2004 | *N* = 185<br>**Age:**<br>5–10: 55.1%<br>11–16: 44.9%<br>**Gender:**<br>Male: 46.5%<br>Female: 53.5%<br>**Race and ethnicity:**<br>African American: 33.5%<br>White: 28.6%<br>Latino: 37.8% |
| Villagrana et al., 2017 | To explore racial and ethnic differences in mental health service utilization among youth while in foster care and at exit | Linked administrative child welfare agency data and public mental health system data | *N* = 1,715<br>**Age:**<br>17: 59%<br>18: 28.7%<br>19: 9.9%<br>20: 2.5%<br>**Gender:**<br>Male: 45.1%<br>Female: 54.9%<br>**Race and ethnicity:**<br>African American: 42.9%<br>White: 16.2%<br>Latino: 40.9% |
| Yampolskaya et al., 2017 | To explore the parallel trajectories of mental health service use and mental health status among children in out-of-home care | Administrative state child welfare data and Medicaid claims in Florida during fiscal year 2007 through fiscal year 2010 | *N* = 6,847<br>**Age:**<br>*M* = 9.54 (*SD* = 4.54)<br>**Gender:**<br>Male: 51%<br>**Race and ethnicity:** |

| | | | |
|---|---|---|---|
| | | | Caucasian: 42%<br>African American: 40%<br>Hispanic: 11%<br>Other: 7% |
| Zima et al., 2000 | To present attempts to access and patterns of service-use among school-aged children in foster care, including moderating factors | Primary data collected through telephone interviews | *N* = 472<br>**Age:**<br>*M* = 8.1<br>**Gender:**<br>Male: 58%<br>Female: 42%<br>**Race and ethnicity:**<br>Caucasian: 17%<br>African American: 42%<br>Latino: 25%<br>Biracial: 12%<br>Other: 3% |
